# Achieving Over 30% Body Weight Loss With Semaglutide in a Patient: A Case Report

**DOI:** 10.7759/cureus.79254

**Published:** 2025-02-18

**Authors:** Sidra Danish, Vallabh Dogra, Uzma Rauf, Maryam Saghir, Nadia Aslam

**Affiliations:** 1 Internal Medicine, Bon Secours Mercy Health, Toledo, USA

**Keywords:** hypertension, hypertension and therapy, prediabetes treatment, semaglutide, weight loss

## Abstract

A 69-year-old female with morbid obesity (BMI: 47) and hypertension achieved significant weight loss following treatment with semaglutide. Starting at 241 lb, she lost 90 lb (over 30% of her body weight) over 65 weeks without side effects. This weight loss exceeded the average reduction reported in previous studies. Additionally, her hypertension resolved, and her glucose levels normalized, illustrating the multifaceted benefits of semaglutide in weight management.

## Introduction

Semaglutide is well-recognized for its role in promoting weight loss via various mechanisms. It activates glucagon-like peptide-1 (GLP-1) receptors in the hypothalamus, which regulate both hunger and satiety, and patients often report reduced appetite and increased satiety, leading to lower caloric consumption. Semaglutide also delays gastric emptying, which helps with satiety and reduces postprandial blood glucose spikes. More importantly, semaglutide has a metabolic effect by increasing insulin secretion and reducing glucagon release, thereby enhancing insulin sensitivity [1]. However, the extent of weight reduction varies among individuals based on the duration of treatment, medication tolerance, the presence of other metabolic comorbidities, and the use of medications that could potentially lead to weight gain (e.g., steroids). Previous studies have shown an average weight loss of 15% over 68 weeks with 2.4 mg weekly doses [2]. This case demonstrates that semaglutide can lead to substantial weight loss exceeding 30% at a dosage of 2.0 mg/week, which is lower than the dose tested in clinical trials.

## Case presentation

A 69-year-old female with a history of primary hypertension, prediabetes, and morbid obesity (BMI: 47) faced significant limitations in ambulation due to her weight. Despite lifestyle modifications, which mainly included dietary changes and, to some extent, physical therapy, which was limited by her morbid obesity, she failed to achieve meaningful weight loss, prompting consideration of semaglutide as an alternative to bariatric surgery. No other weight loss medications were tried before starting semaglutide.

**Figure 1:**
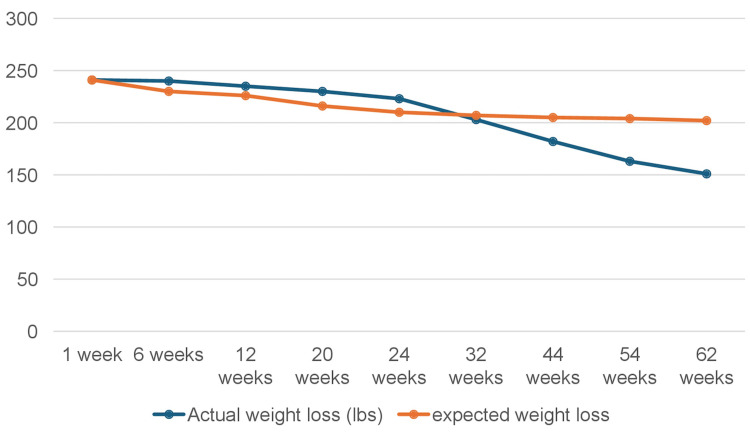
Observed vs. expected weight loss with semaglutide in clinical trials

The patient began semaglutide at 0.25 mg/week for four weeks, followed by 0.5 mg/week for another four weeks. The dose was then increased to 1 mg/week and subsequently to 2 mg/week, as reported in the medication guidelines [3]. The patient was assessed at regular intervals for medication tolerance and to rule out adverse effects such as gastrointestinal issues, fatigue, reduced appetite, and pancreatitis, which have been cited as reasons for discontinuation in other studies [4]. The patient reported no adverse effects throughout 65 weeks of treatment. She experienced a remarkable weight reduction of 90 lb, equating to 37.34% of her initial body weight, in contrast to the 15% reduction reported in previous studies (Figure 1) [2,5]. Concurrently, her hypertension improved to the point that all antihypertensive medications (lisinopril and hydrochlorothiazide) were discontinued, and she maintained normotension throughout treatment. Additionally, her prediabetes resolved, with hemoglobin A1c returning to the normal range.

To exclude other causes of significant weight loss, an extensive evaluation was performed [6]. A thorough clinical history was taken again, and it was not suggestive of any underlying cause of unintentional weight loss. Her thyroid profile was unremarkable. The patient was up to date with cancer screenings, including mammography and colonoscopy. She had undergone a total hysterectomy with cervix removal, eliminating the need for cervical cancer screening. A CT scan of the chest, abdomen, and pelvis ruled out occult malignancy, further supporting that the weight loss was attributable to semaglutide therapy.

## Discussion

This case underscores the significant weight loss potential of semaglutide and its associated health benefits. Weight loss is associated with remarkable beneficial outcomes, including improved insulin sensitivity, lower blood pressure, better glycemic control and dyslipidemia, decreased systemic inflammation and endothelial dysfunction, reduced progression of non-alcoholic fatty liver disease (NAFLD), and a lower risk of cardiovascular events and mortality [6]. All these beneficial effects are linearly related to the extent of weight loss. While clinical trials report an average weight loss of approximately 15% with 2.4 mg weekly doses of semaglutide over 68 weeks [2,5,7], this patient achieved a 37.34% weight reduction with a lower dose of 2.0 mg/week. This suggests that greater weight loss may be achievable in select individuals when the medication is well-tolerated and used for a longer duration.

The patient's case highlights semaglutide's role in addressing obesity-related comorbidities, including hypertension and prediabetes. Other potential benefits of weight loss, such as a reduction in cardiometabolic risk factors, further demonstrate the broad therapeutic impact of this treatment [8].

It is essential to rule out alternative causes of weight loss, such as malignancy, gastrointestinal, and endocrine pathologies, when observing significant reductions in body weight. This patient’s comprehensive evaluation confirmed that her weight loss was associated with semaglutide therapy.

## Conclusions

This case shows that the response to semaglutide is dose-dependent and varies based on patient-specific factors. The patient was able to lose more than 30% of her body weight with semaglutide, far exceeding the clinical trial results of approximately 15%. This demonstrates that individual responses to semaglutide may vary, and further research is needed to evaluate the factors associated with significant weight loss. The patient's remarkable weight reduction and resolution of her comorbidities highlight the transformative potential of this medication, supporting its use as an effective intervention for significant weight loss and the management of obesity-related comorbidities.
